# Characterizing Individuals Fulfilling Clinical Criteria for Limbic-Predominant Age-Related TDP-43 Encephalopathy in a Tertiary Memory Clinic

**DOI:** 10.1212/WNL.0000000000214810

**Published:** 2026-04-16

**Authors:** Colin Groot, Ismael L. Calandri, Ilse Bader, Diana I. Bocancea, Hannah de Bruin, Maria Carrigan, Lyduine E. Collij, Flora H. Duits, Suzie Kamps, Lotte A. de Koning, Afina W. Lemstra, Sophie E. Mastenbroek, Roos M. Rikken, Bastiaan G.J. van Tol, Marie R. Vermeiren, Alex Wesseling, Ye Xia, Charlotte E. Teunissen, Elsmarieke van de Giessen, Frederik Barkhof, Laura E. Jonkman, Casper de Boer, Annemieke J. Rozemuller, Anna E. Leeuwis, Annelies E. van der Vlies, Betty M. Tijms, Wiesje M. van der Flier, Yolande A.L. Pijnenburg, Emma M. Coomans, Rik Ossenkoppele

**Affiliations:** 1 Alzheimer Center Amsterdam, Neurology, Vrije Universiteit Amsterdam, Amsterdam UMC, the Netherlands;; 2 Amsterdam Neuroscience, Neurodegeneration, the Netherlands;; 3 Department of Cognitive Neurology, Fleni, Buenos Aires, Argentina;; 4 Faculty of Science, Swammerdam Institute for Life Sciences, University of Amsterdam, the Netherlands;; 5 Radiology & Nuclear Medicine, Vrije Universiteit Amsterdam, Amsterdam UMC, the Netherlands;; 6 Clinical Memory Research Unit, Lund University, Sweden;; 7 Amsterdam UMC, Department of Anatomy and Neurosciences, Location Vrije Universiteit Amsterdam, the Netherlands;; 8 Gordon Center for Medical Imaging, Massachusetts General Hospital, Boston;; 9 Harvard Medical School, Boston, MA;; 10 Neurochemistry Laboratory, Department of Laboratory Medicine, Vrije Universiteit Amsterdam, Amsterdam UMC location VUmc, the Netherlands;; 11 Queen Square Institute of Neurology and Centre for Medical Image Computing, University College London, United Kingdom;; 12 Epidemiology & Data Science, Vrije Universiteit Amsterdam, Amsterdam UMC location VUmc, the Netherlands;; 13 Alzheimer Nederland, Amersfoort, the Netherlands; and; 14 Amsterdam Public Health, the Netherlands.

## Abstract

**Background and Objectives:**

Limbic-predominant age-related TDP-43 encephalopathy (LATE) is characterized by an amnestic- and limbic-predominant phenotype, which can mimic Alzheimer disease (AD). In a memory clinic cohort, we tested whether clinical criteria for LATE can detect a clinical profile of LATE that is distinct from AD.

**Methods:**

In this retrospective examination of a longitudinal memory clinic cohort from the Alzheimer Center Amsterdam, we included individuals with mild cognitive impairment (MCI) and dementia (aged >50 years). We classified individuals based on baseline data on cognition, atrophy, amyloid-status, and tau-status into Probable- and Possible-LATE, co-occurring LATE and AD (LATE-AD), and AD (without LATE). Next, we compared these groups on demographics, clinical features, cognition, and atrophy.

**Results:**

Of 3,606 individuals (mean age at baseline 66 [SD 6], 49.2% female) available for classification, we classified 56 (1.6%) as Probable-LATE, 115 (3.2%) as Possible-LATE, 127 (3.5%) as LATE-AD, and 1,675 (46.5%) as AD. Individuals with Probable-LATE progressed slower than AD on mini-mental state examination (MMSE) (sβ [SE] = 0.12 [0.05], *p* = 0.02), memory (sβ [SE] = 0.11 [0.5], *p* = 0.01), attention (sβ [SE] = 0.12 [0.16], *p* = 0.05), executive functioning (sβ [SE] = 0.09 [0.04], *p* = 0.03), and visuospatial functioning (sβ [SE] = 0.10 [0.05], *p* = 0.05). Individuals with LATE-AD progressed faster than AD on MMSE (sβ [SE] = −0.12 [0.05], *p* = 0.01), attention (sβ [SE] = −0.13 [0.06], *p* = 0.04), and executive functioning (sβ [SE] = −0.10 [0.05], *p* = 0.03). Mortality risk, compared to AD, was lower in individuals with Probable-LATE (hazard ratio [HR] 0.70 [0.49–0.99], *p* = 0.04) and higher in Possible LATE-AD (HR 1.25 [1.01–1.53], *p* = 0.04). Compared to AD, at baseline, individuals with Probable-LATE and Possible-LATE had higher inferior temporal-to-hippocampus ratios (indicating limbic-predominant atrophy; sβ [SE] = 0.59 [0.16], *p* < 0.01; sβ [SE] = 0.40 [0.13], *p* < 0.01), and Probable-LATE, Possible-LATE, and LATE-AD all showed smaller amygdalar volumes at baseline than AD (sβ [SE] = −0.55 [0.15], *p* < 0.01; sβ [SE] = −0.43 [0.12], *p* < 0.01; sβ [SE] = −0.62 [0.11], *p* < 0.01). Individuals with LATE-AD had thinner cortex at baseline in an “AD-signature” composite region compared to AD (sβ [SE] = −0.73 [0.11], *p* < 0.01).

**Discussion:**

Using an operationalization of clinical criteria for LATE, 8.2% of participants with MCI or dementia from our tertiary memory clinic were classified as Possible-LATE, Probable-LATE, or LATE-AD. Probable-LATE was characterized by a milder disease course than AD, whereas LATE-AD was characterized by a more aggressive disease course. This underscores the value of the proposed clinical criteria in identifying individuals with suspected LATE, who have distinct clinical trajectories from AD. Our findings, therefore, support the use of these criteria to improve diagnostic and prognostic accuracy in the memory clinic.

## Introduction

Limbic-predominant age-related TDP-43 encephalopathy (LATE) is a pathologic entity that is associated with the pathologic finding of TAR DNA-binding protein 43 (TDP-43) proteinopathy in limbic brain regions,^1^ a finding that is termed LATE neuropathologic change (LATE-NC). During life, individuals with LATE-NC typically display an amnestic profile that mimics the early stages of Alzheimer disease (AD), which complicates clinical differentiation between LATE and AD.^2^ This is reinforced by the radiologic features of LATE, that is, limbic-predominant atrophy, which is also characteristic of (early) AD.^3,4^ This neurobiological fingerprint closely expresses the localization of pathology in both LATE (TDP-43 proteinopathy) and early AD (neurofibrillary tau tangles), which both have a propensity for limbic brain regions.^1,5^ Although intracellular TDP-43 inclusions and their harmful effects on the brain have been recognized for some years,^6^ TDP-43 proteinopathy was, at first, more commonly associated with frontotemporal lobar degeneration and amyotrophic lateral sclerosis. More recently, the high prevalence of LATE-NC and its clinical consequences have become apparent based on assessments in clinico-pathologic cohorts.^7-9^

LATE-NC often co-occurs with AD pathology, especially with advancing age.^1,9-12^ The co-occurrence of LATE-NC and AD pathology acts synergistically and results in worse clinical outcomes than isolated AD pathology, whereas LATE-NC without AD pathology seems to result in slower clinical progression than “pure” AD.^1,8,9,11,13,14^ Although clinically similar, differences between the clinical expressions of LATE and AD have been reported. For example, LATE is characterized by a sustained amnestic-predominant profile until later stages, whereas individuals with AD generally progress to multidomain impairment faster.^8^

To date, there are no established biomarkers with molecular specificity for TDP-43 pathology. Therefore, expert consensus criteria were recently proposed that provide guidelines for clinically diagnosing LATE.^10^ These criteria are based on the key characteristics of LATE, that is, an amnestic clinical profile and a limbic-predominant neurodegeneration pattern, in conjunction with excluding AD based on biomarkers (i.e., β-amyloid [Aβ] and tau).^10^ This results in groups of (Probable and Possible) LATE, co-occurring LATE and AD (i.e., Possible LATE-AD), and AD (without LATE).

In this study, our objective was to operationalize the proposed clinical criteria for LATE and use them to identify groups of Probable/Possible LATE and LATE-AD in a tertiary memory clinic cohort of individuals with mild cognitive impairment (MCI) or dementia, closely resembling the intended real-world applicability of these criteria. We hypothesize that applying the proposed clinical criteria for LATE yields groups of individuals who have distinct clinical and atrophy trajectories compared to a reference group of individuals with symptomatic AD, which would highlight the utility of these criteria in aiding prognostication.

## Methods

### Participants

We used data collected between September 1997 and March 2024 from the Amsterdam Dementia Cohort (ADC), established by the Alzheimer Center Amsterdam. Detailed study procedures are outlined in the eMethods and have also been described previously.^15,16^ In brief, the Alzheimer Center Amsterdam functions as a tertiary memory clinic that receives referrals from neurologists, geriatricians, psychiatrists, and general practitioners from throughout the Netherlands. Individuals are often referred for second opinions. All patients undergo a diagnostic workup that includes neuropsychological testing, brain MR, and lumbar puncture, and are included in the ADC if they give informed consent to use their data for research purposes.

Positivity for Aβ was determined by Aβ-PET visual read or CSF. Tau positivity (T+) was determined by ptau181 in CSF or, in a limited number of cases (n = 52), [^18^F]flortaucipir-PET visual read. For the present study, we only selected individuals who were >50 years of age with cognitive impairment (i.e., a syndrome diagnosis of MCI or dementia [Table 1]^17-24^). To be eligible for classification into our target groups, baseline neuropsychological test data needed to be sufficient to determine amnestic or multidomain impairment (see “Cognition” section). Furthermore, to determine a limbic-predominant radiologic phenotype, individuals needed to have a baseline MRI scan that was of sufficient quality for visual assessment of medial temporal atrophy (MTA),^4,25^ parietal atrophy (PA), and global cortical atrophy (GCA)^26^ to determine a limbic-predominant atrophy pattern (see “MRI” section).

**Table 1:** Demographics Across Groups

	Probable LATE (N = 56)	Possible LATE (N = 115)	LATE-AD (N = 127)	AD (N = 1,675)	*p* Value
Age, y	66.00 (6.38)	69.86 (6.23)	70.14 (6.77)	66.07 (7.08)	*F* = 22.56, *p* < 0.001
Female, n (%)	18 (32.1)	60 (52.2)	57 (44.9)	835 (49.9)	χ^2^ = 8.16, *p* = 0.043
Education^a^	4.69 (1.17)	5.09 (1.24)	5.09 (1.34)	5.12 (1.28)	χ^2^ = 8.16, *p* = 0.043
MMSE	24.13 (3.10)	21.70 (4.11)	19.00 (5.55)	22.58 (4.94)	*F* = 24.12, *p* < 0.001
NPI total	11.80 (11.23)	14.53 (15.56)	13.11 (13.54)	11.56 (12.25)	*F* = 1.89, *p* = 0.129
*APOE* ε4, n (%) carrier	20 (40.0)	60 (66.7)	86 (74.1)	1,128 (71.8)	χ^2^ = 21.44, *p* < 0.001
Estimated symptom duration^b^	3.87 (3.51)	3.84 (3.65)	3.22 (1.94)	3.13 (2.51)	*F* = 3.86, *p* = 0.009
Syndrome diagnosis, MCI	16 (28.6)	8 (7.0)	6 (4.7)	458 (27.3)	χ^2^ = 53.41, *p* < 0.001
Suspected etiology^c^					χ^2^ = 544.97, *p* < 0.001
AD	20 (50.0)	96 (89.7)	115 (95.0)	1,217 (100.0)	
Dementia not specified	3 (7.5)	1 (0.9)	0 (0.0)	0 (0.0)	
DLB	2 (5.0)	1 (0.9)	0 (0.0)	0 (0.0)	
FTD	15 (37.5)	4 (3.7)	6 (5.0)	0 (0.0)	
VaD	0 (0.0)	5 (4.7)	0 (0.0)	0 (0.0)	
CSF Aβ42, Innotest	1,120.89 (210.51)	577.30 (114.21)	591.45 (92.10)	619.59 (108.52)	*F* = 293.6, *p* < 0.001
CSF Aβ42, Elecsys	1,368.25 (210.59)	549.12 (165.07)	553.87 (140.25)	602.69 (182.69)	*F* = 48.7, *p* < 0.001
CSF ptau, Innotest	54.03 (26.25)	41.69 (11.26)	93.56 (35.37)	88.06 (38.80)	*F* = 30.43, *p* < 0.001
CSF ptau, Elecsys	22.46 (7.79)	16.69 (9.21)	33.79 (10.32)	35.94 (19.05)	*F* = 6.6, *p* < 0.001
CSF total tau, Innotest	582.54 (1,183.23)	315.14 (103.37)	761.50 (355.43)	708.61 (412.81)	*F* = 10.17, *p* < 0.001
CSF total tau, Elecsys	260.57 (65.82)	182.53 (69.19)	344.72 (84.98)	352.13 (168.30)	*F* = 6.2, *p* < 0.001

Abbreviations: Aβ = β-amyloid; AD = Alzheimer disease; APOE = apolipoprotein E; DLB = Lewy body dementia; FTD = frontotemporal dementia; IQR = interquartile range; LATE = limbic-predominant age-related TDP-43 encephalopathy; MCI = mild cognitive impairment; MMSE = mini-mental state examination; NPI = neuropsychiatric inventory; ptau = hyperphosphorylated tau; VaD = vascular dementia.

Values are presented as n with the proportion within each group (%) for categorical variables and means with their SD within each group for continuous variables. *p* Values for overall group differences obtained using analysis of variance, Chi-squared, and Kruskal Wallis tests (applied where appropriate). CSF measures were performed using 2 different assays: Innotest before June 2018 and Elecsys after (eMethods). Pairwise differences between all groups are displayed in eTable 5. Missing data; education n = 16; MMSE n = 35; NPI = 326; APOEε4 status n = 146; symptom duration = 52; CSF amyloid = 710; CSF ptau = 650; CSF total tau = 650.

a Measured on the qualitative Verhage scale from 1 to 7 with 1 indicating not having finished primary school, and 7 indicating a university degree.^27^

b Based on patient or caregiver self-report.

c Only in dementia cases and based on multidisciplinary consensus.

### Cognitive Evaluation

Included individuals underwent a comprehensive neuropsychological test battery at baseline and follow-up visits. Test scores were categorized into 5 cognitive domains (memory, attention, language, executive functioning, and visuospatial ability).^28^ Included tests per domain are reported in eMethods. As a measure of global cognitive functioning, we implemented mini-mental state examination (MMSE) scores. To provide an estimate of global cognitive decline before diagnosis, we additionally computed an MMSE yearly decline metric that is obtained by subtracting the MMSE contemporaneous to dementia screening from 30 (max score for the MMSE) and then dividing by the self-reported symptom duration. Further details on processing of neuropsychological data are outlined in eMethods.

### Operationalization of LATE Criteria and Group Classification

**Figure 1:**
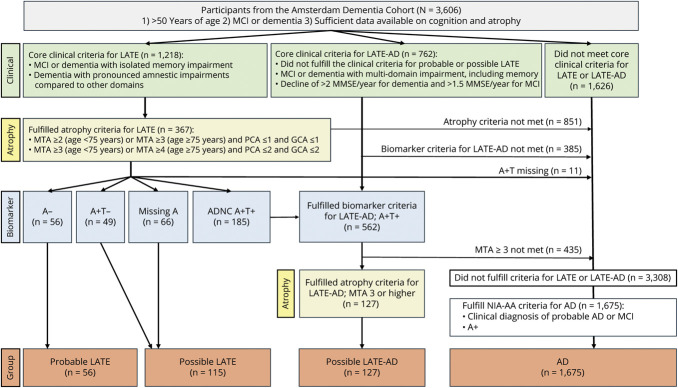
Flowchart of Participant Classification Steps A = amyloid-β biomarkers; AD = Alzheimer disease; GCA = global cortical atrophy; LATE = limbic-predominant age-related TDP-43 encephalopathy; MCI = mild cognitive impairment; MTA = medial temporal atrophy; NIA-AA = National Institute on Aging-Alzheimer's Association; PA = posterior atrophy; T = tau biomarkers.

The consensus criteria outlined by Wolk et al.,^10^ classify individuals into 3 separate LATE groups in a probabilistic manner based on whether they are suspected to have LATE-NC using clinical, radiologic, and biomarker profiles. Probable LATE has a clinical-radiological profile fulfilling LATE criteria with negative amyloid-biomarkers. This group is deemed most likely to have LATE-NC as the driving etiology (i.e., “pure LATE”). Possible LATE has a clinical-radiological profile fulfilling LATE criteria with positive or missing amyloid biomarkers. Possible LATE-AD has a clinical-radiological profile fulfilling LATE criteria in addition to positive amyloid- and tau-biomarkers (i.e., suspected LATE-NC/AD neuropathologic change co-pathology). The specific required and supporting criteria for every one of these categories are outlined in eTables 1 and 2, and in the original publication.^10^ In this study, the consensus criteria outlined by Wolk et al.^10^ were adapted to accommodate the population and data availability in the ADC. The specific operationalization of each of the criteria is detailed in eTables 1 and 2, and in Figure 1. Briefly, an individual was classified as LATE when there was an isolated memory impairment, or, for dementia cases only, a cognitive profile that was characterized by a disproportionate memory impairment relative to the other cognitive domain scores, that is, memory is more than 1SD below the average across all domains. The latter accounts for a possible amnestic-predominant but multidomain impairment in later LATE disease stages. A radiologic phenotype fitting with LATE was defined by age-adjusted visual read measures of atrophy determined on structural MRI: MTA, PA, and GCA.^4,26^ Consistent with criteria for AD, the radiologic criteria for LATE do not define a strict cutoff when it comes to classifying a limbic-predominant atrophy profile. In our operationalization, individuals could have (1) MTA ≥2 (when aged <75) or MTA ≥3 with PA and/or GCA ≤1 or (2) MTA ≥3 (aged >75) or MTA ≥4 with GCA and/or PA 2. As outlined in the Wolk et al. publication,^10^ among individuals who adhered to these clinical and radiologic profiles, those with negative amyloid-biomarkers (i.e., A−) were categorized as “Probable LATE.” Individuals with missing A biomarkers (A_missing_) or with an A+T− biomarker profile were categorized as “Possible LATE”. The individuals who adhered to the clinical and radiologic profiles for LATE but who were A+T+ (and, therefore, not eligible to be classified as Probable or Possible LATE) were pooled with A+T+ individuals with a multidomain impairment (including memory) for further classification. Among this combined group, those with MTA ≥3 were classified as “Possible LATE-AD,” henceforth referred to as “LATE-AD” to avoid confusion with the Possible LATE group. A+T+ individuals who did not meet the MTA ≥3 criterion for LATE-AD were grouped with all A+ individuals who did not meet clinical and radiologic profiles for any of the LATE groups and were classified into the AD reference group if they also received a clinical diagnosis of AD based on a multidisciplinary consensus meeting. The flowchart in Figure 1 describes the participant classification steps in greater detail.

### Mortality

Survival was defined as the time from the screening visit at the Alzheimer Center Amsterdam to the event (being either death or censoring). Data on mortality were collected from the Central Public Administration until September 2024 and included the status (dead/alive) and date of death. If data from the Central Public Administration were not available, the date of the last visit to the Alzheimer Center Amsterdam was included as the censoring date.

### Neuroimaging Processing

We acquired structural T1-weighted MR images according to standardized acquisition protocols described previously.^29^ Regional cortical thickness from regions of the Desikan-Killiany atlas were obtained in subject-space using FreeSurfer (version 7.1),^30^ and data were harmonized across scanners using neurocombat.^31^ To cover regions typically associated with LATE or AD (or both), we selected the following regions-of-interest (ROIs) to assess hippocampal volume, amygdalar volume, entorhinal thickness, AD signature cortical thickness (weighted average of entorhinal, inferior temporal, middle temporal, parahippocampal, rostral middle frontal, medial orbitofrontal, precuneus, inferior parietal, and supramarginal cortical thickness), and a ratio between inferior temporal thickness/hippocampal volume (ITH ratio; smaller values indicate relative limbic vs neocortical atrophy), which has been proposed to distinguish LATE from AD.^32-34^ We will present results for the AD signature ROI, amygdalar ROI, and the ITH ratio in the main text, and the rest of the ROIs are displayed in the supplemental material.

### Statistical Analyses

All statistical analyses and visualizations were performed in R software (version 4.3.3).

We fitted linear regression models to examine cross-sectional differences between groups on cognition and atrophy, and we fitted linear mixed models (LMMs) to examine differences in longitudinal trajectories in cognitive domains scores and brain atrophy. Models were adjusted for age, sex, and education (for models with cognition as the outcome). For LMM, we fitted models with and without random slopes and intercepts and selected the best-fitting model based on the lowest Akaike Information Criterion value. Cox proportional-hazard models were performed to evaluate whether differences in mortality risk were statistically significant when correcting for the potential confounders age, sex, education, and syndrome diagnosis. The Cox hazard model assumption of proportionality of effects across groups over time was assessed using Schoenfeld residuals, and log-log plots.^35^

Across all analyses, we will focus on differences between the LATE groups and AD in the main text, whereas pairwise differences between all groups are outlined in the supplement.

### Standard Protocol Approvals, Registrations, and Patient Consents

Written informed consent was obtained for participation in the ADC, and study procedures were approved by the institutional review board of the Amsterdam UMC (REC 2017.315).

### Data Availability

Anonymized data supporting the findings of this study are accessible to a qualified investigator upon reasonable request.

## Results

After applying our inclusion and exclusion criteria (>50 years with MCI or dementia), we arrived at a sample of 4,042 individuals. Of these 4,042 individuals, 88 (2.2%) did not have sufficient cognitive evaluation data available to determine a clinical profile. Of the remaining 3,954 individuals, 348 (8.8%) did not have sufficient MRI visual read data available to determine atrophy profiles. Therefore, a total of 3,606 individuals (mean age at baseline 66 [SD 6], 49.2% female) were eligible for classification. Amyloid-data were available for 2,856/3,606 individuals (79.2%; 775 negative and 2,081 positive). Tau data were available for 2,715/3,606 individuals (75.3%; 872 negative and 1,843 positive).

Following the participant classification steps outlined in Figure 1, we classified 56/3,606 (1.6% of all eligible individuals) as Probable LATE, 115 (3.2%) as Possible LATE, and 127 (3.5%) as LATE-AD, totaling 298 (8.2%) individuals being classified into one of the clinical LATE groups. Of the 3,308 participants who did not fulfill criteria for Probable LATE, Possible LATE, or LATE-AD, 1,675 participants (46.5%) were amyloid-positive and had a syndrome diagnosis of MCI or probable AD and were thereby categorized into our AD reference group. The remaining 1,633 (45.3%) individuals did not meet our criteria for either LATE or AD and were not included in the rest of our analyses. Table 1 displays the demographic and clinical characteristics of the groups, and eTables 3 and 4, and eFigure 1, display an overview of LATE features observed across age ranges (50–60, 60–70, 70–80, and 80+) and between men and women. All pairwise differences between groups are given in eTable 5, and the differences between the LATE groups and AD are described here. The Probable LATE group had a higher percentage of women and a lower prevalence of *APOE* ε4 compared to AD. The Possible LATE and LATE-AD groups were older than the AD group, and the LATE-AD group additionally had a lower baseline MMSE than AD (Table 1; eTable 5).

### Cognition

**Figure 2:**
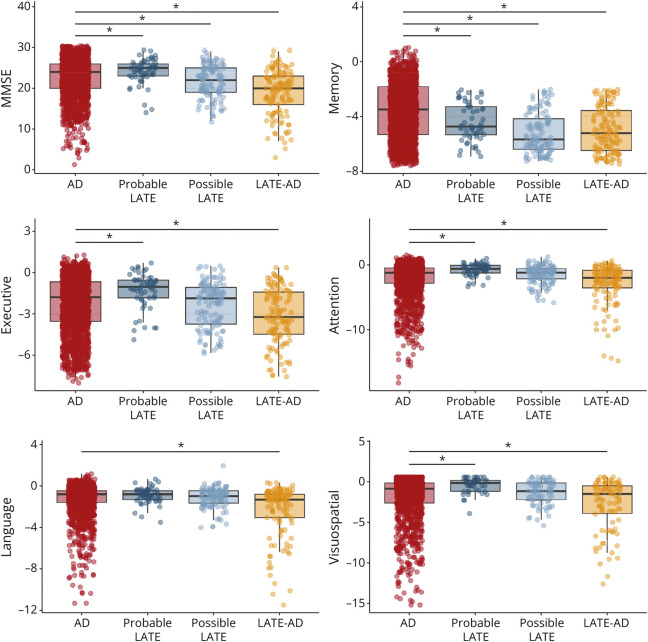
Baseline Cognitive Performance Across Diagnostic Groups Differences between AD and the LATE groups in baseline cognition are indicated by the significance bars. All pairwise differences between groups are outlined in eTable 6. AD = Alzheimer disease; LATE = limbic-predominant age-related TDP-43 encephalopathy; MMSE = mini-mental state examination.

Group differences described here will focus on differences between AD and the LATE groups, all pairwise differences between groups are provided in eTable 6. Because baseline cognitive scores were part of our group classification, differences in baseline cognitive scores between groups should be regarded as descriptive and are only outlined in detail in Figure 2 and eTable 6.

**Figure 3:**
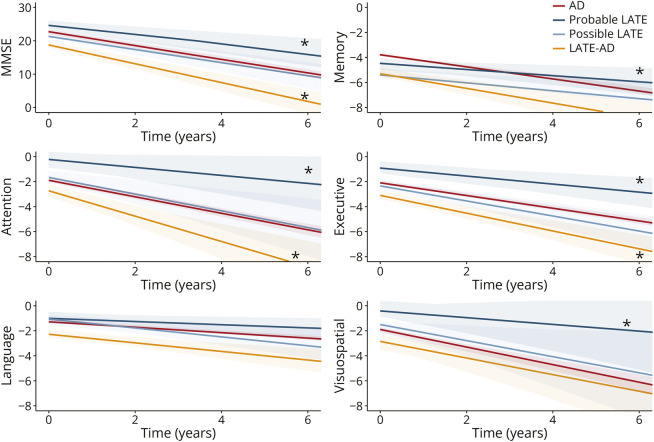
Longitudinal Cognitive Performance Across Diagnostic Groups Differences between AD and the LATE groups in longitudinal cognitive performance (indicated by the slope) are indicated by an *. All pairwise differences between groups are outlined in eTable 6. AD = Alzheimer disease; LATE = limbic-predominant age-related TDP-43 encephalopathy; MMSE = mini-mental state examination.

For longitudinal decline, we observed that individuals with Probable LATE progressed slower than AD on MMSE (sβ [SE] = 0.12 [0.05], *p* = 0.02), memory (sβ [SE] = 0.11 [0.05], *p* = 0.01), attention (sβ [SE] = 0.12 [0.06], *p* = 0.05), visuospatial functioning (sβ [SE] = 0.10 [0.05], *p* = 0.05), and executive functioning (sβ [SE] = 0.09 [0.04], *p* = 0.03). Furthermore, individuals with LATE-AD progressed faster than AD on MMSE (sβ [SE] = −0.12 [0.05], *p* = 0.01), attention (sβ [SE] = −0.13 [0.06], *p* = 0.04), and executive functioning (sβ [SE] = −0.10 [0.05], *p* = 0.03). There were no differences in longitudinal cognitive performance between individuals with Possible LATE and AD (Figure 3).

### Mortality

**Figure 4:**
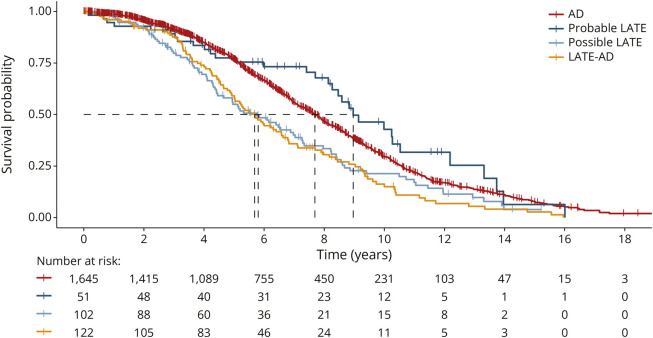
Mortality Rates Across Diagnostic Groups All pairwise differences between groups in HR are displayed in eTable 7. AD = Alzheimer disease; HR = hazard ratio; LATE = limbic-predominant age-related TDP-43 encephalopathy.

Out of the 1,973 individuals included in the main analyses, 1,187 (60.2%) died over the course of follow-up (mean follow-up time 6.0 [3.6]). The estimated median survival time was 9.0 years (95% CI 8.3–12.2) for individuals with Probable LATE, 7.7 years (7.4–8.0) for AD, 5.8 years (4.7–7.3) for individuals with Possible LATE, and 5.7 years (5.0–6.6) for individuals with LATE-AD (Figure 4). Cox proportional-hazards models showed that mortality risk, compared to AD, was lower in individuals with Probable LATE (hazard ratio [HR] 0.70 [0.49–0.99], *p* = 0.04), whereas mortality risk was higher in the LATE-AD group (HR 1.25 [1.01–1.53], *p* = 0.04). There was no difference in mortality risk between individuals with Possible LATE and AD (HR 1.17 [0.93–1.47], *p* = 0.17; Figure 4). All pairwise differences between LATE groups are displayed in eTable 7. Assessment of Schoenfeld residuals and log-log plots indicated that the assumption of proportionality of group effects over time was met (eFigure 2).

### MRI

Group differences described here will again focus on differences between AD and the LATE groups; all pairwise differences between groups are provided in eTable 8, and results from additional regions of interest are displayed in eFigure 3.

Individuals with LATE-AD had thinner cortex in an “AD-signature” composite region compared to AD at baseline (sβ [SE] = −0.73 [0.11], *p* < 0.01), indicating a greater degree of cortical atrophy. Individuals with Probable LATE, Possible LATE, and LATE-AD all showed smaller amygdalar volumes at baseline than AD (sβ [SE] = −0.55 [0.15], *p* < 0.01; sβ [SE] = −0.43 [0.12], *p* < 0.01; sβ [SE] = −0.62 [0.11], *p* < 0.01).

**Figure 5:**
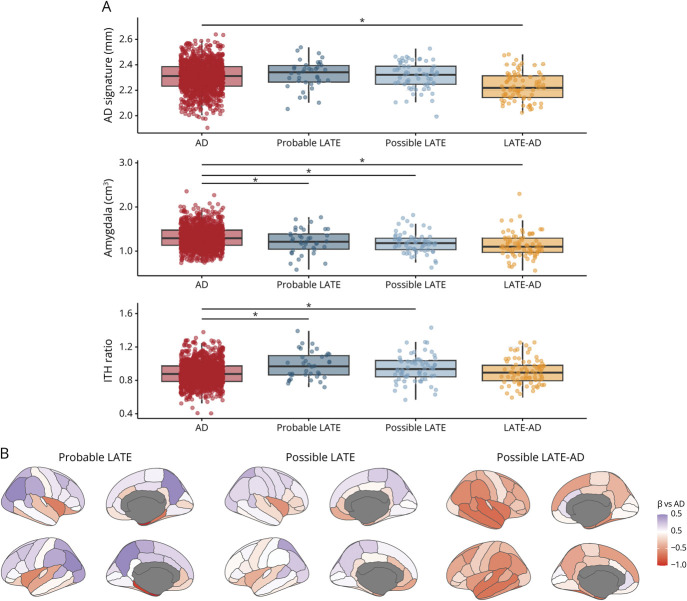
Baseline Brain Atrophy Across Diagnostic Groups (A) Bar graph showing group differences in baseline atrophy. Differences between AD and the LATE groups obtained using general linear models are indicated by the significance bars. (B) The surface projections are colored according to the β of the group effects on atrophy (i.e., LATE groups vs AD) from general linear models assessing cross-sectional atrophy. All pairwise differences between groups are outlined in eTable 8, and results from additional regions of interest are displayed in eFigure 3. AD = Alzheimer disease; ITH = inferior temporal thickness/hippocampus volume, higher values indicate more limbic-predominant atrophy; LATE = limbic-predominant age-related TDP-43 encephalopathy.

Individuals with Probable LATE and Possible LATE had higher inferior temporal to hippocampus ratios (indicating limbic-predominant atrophy) compared to AD (sβ [SE] = 0.59 [0.16], *p* < 0.01; sβ [SE] = 0.40 [0.13], *p* < 0.01; Figure 5). Note that relative medial temporal atrophy was an inclusion criterion for the LATE groups, so these results were expected.

**Figure 6:**
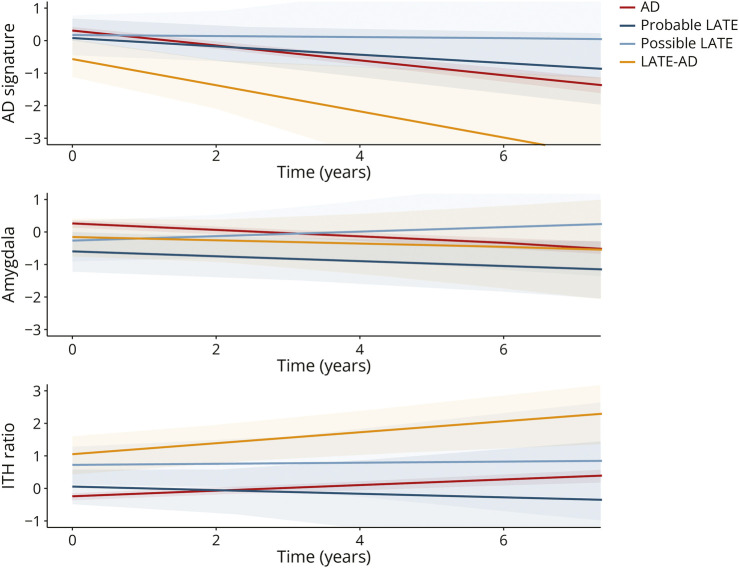
Longitudinal Brain Atrophy Across Diagnostic Groups Line graphs indicate differences in longitudinal atrophy between groups. Differences between AD and the LATE groups obtained using linear mixed effects analyses (indicated by the slope) are highlighted with an *. Although the Possible and Probable LATE groups tended to have a slower decline in AD-signature region cortical thickness (top graph), none of the time × group interaction effects were significant (*p* > 0.05). All pairwise differences between groups are outlined in eTable 8, and results from additional regions of interest are displayed in eFigure 3. AD = Alzheimer disease; ITH = inferior temporal thickness/hippocampus volume, higher values indicate more limbic-predominant atrophy; LATE = limbic-predominant age-related TDP-43 encephalopathy.

We did not observe any significant differences in longitudinal atrophy rates between the groups (all *p* > 0.05; Figure 6).

### Sensitivity Analyses

The Possible LATE group consisted of individuals with an A+T− biomarker profile (N = 49) and those with missing A biomarkers (A_missing_; N = 66). To assess possible differences between these 2 subgroups in our primary outcomes, we separated these subgroups and assessed longitudinal cognition (eTable 9) and mortality rates (eTable 10). We found that individuals with Possible LATE A+T− had a milder decline in visuospatial functioning (sβ [SE] = 0.32 [0.10], *p* < 0.01). We also observed differences between Possible LATE A+T− and Possible LATE A_missing_ groups in terms of contrasts with the AD group. In that memory decline was faster for Possible LATE A+T− compared to AD, whereas language decline was slower for Possible LATE A+T− compared to AD.

Similar to the separation of the Possible LATE group, we also separated the Probable LATE group into individuals with an A−T+ profile (N = 21) and compared this group in cognition (eTable 11) and mortality rates (eTable 12) to the rest of the Probable LATE group. We observed that although the Probable LATE T+ group did not differ in longitudinal decline in MMSE, memory, executive functioning, and visuospatial functioning compared to AD, the rest of the Probable LATE group did (all *p* < 0.05). Furthermore, mortality risk was higher in A−T+ Probable LATE compared to the rest of the Probable LATE group (HR 2.83 [1.22–6.55], *p* = 0.02).

We additionally explored models assessing longitudinal cognition, atrophy, and mortality adjusted for *APOE* ε4 carriership and found similar results as reported in the main text (eTable 13), although we did observe that entorhinal cortex atrophy rates were lower in Probable LATE compared to AD (sβ [SE] = 0.91 [0.04], *p* = 0.02) when *APOE* ε4 carriership was adjusted for.

## Discussion

We operationalized the recently proposed consensus clinical criteria for LATE^10^ and adapted them to individuals from a tertiary memory clinic. Based on their clinical, radiologic, and AD biomarker profile, 56 (1.6%) individuals could be classified as Probable LATE, 115 (3.2%) as Possible LATE, and 127 (3.5%) as Possible LATE-AD, whereas 1,675 participants (46.5%) adhered to criteria for AD and were used as a reference group. We observed that individuals with Probable LATE generally showed slower cognitive decline than the groups, and showed lower mortality rates. The LATE-AD group, on the other hand, declined faster on all cognitive domains than AD and had higher mortality rates. We observed that Probable and Possible LATE could be differentiated from AD based on a limbic-predominant atrophy pattern, whereas individuals with LATE-AD showed more severe cortical atrophy compared to AD. Taken together, among a tertiary memory clinic cohort, we were able to classify Possible LATE, Probable LATE, and LATE-AD groups that were not only distinct in their baseline clinical-radiological phenotypes but also showed unique clinical trajectories. These findings highlight that the LATE classification can be highly relevant for diagnostic and prognostic purposes.

The primary challenge in differentiating LATE from AD has been the overlapping clinical and radiologic presentations, characterized by an amnestic-predominant and limbic-predominant profile.^10,12,32,36,37^ The differentiation has been further complicated by a prevalent co-occurrence of LATE-NC and AD pathology, which may be related to shared risk factors or pathologic synergy.^1,38^ In this study, we demonstrate that adapting the newly proposed clinical criteria for LATE and applying them to a tertiary memory clinic cohort produced groups that were distinct from AD in clinical-radiological profiles and clinical trajectories. Among these, Probable LATE was characterized by the mildest clinical decline and lowest mortality rates, which is in line with previous literature.^8,12,37,39,40^ Although other pathologies may still be present to a degree in individuals classified as Probable LATE, LATE-NC is believed to be the dominant pathology.^2,41,42^ In our own assessment, we were unable to confirm underlying TDP-43 pathology due to the limited availability of TDP-43 staining at postmortem assessment. However, given the negative Aβ biomarkers, AD pathology is unlikely to contribute significantly to symptoms in our Probable LATE group. This is further indicated by the relatively low prevalence of *APOE* ε4 (40%) in the Probable LATE group, compared to 70% in AD in our sample, which is an estimate for AD that is in line with previous studies.^43^ Although much more strongly associated with AD pathology, *APOE* ε4 has been reported to be a risk factor for LATE-NC, possibly relating to pathologic synergy with AD.^12,44,45^ The hypothesized association between *APOE* ε4 and LATE-NC might explain why *APOE* ε4 is still over-represented in our LATE groups compared to the general population (25% in Northern-Europe).^43^

A clinical feature that has been consistently associated with LATE-NC is age, and age has even been incorporated into the limbic-predominant age-related TDP-43 encephalopathy (i.e., LATE) term. LATE has been hypothesized to predominantly affect individuals aged 75 and older,^1,46,47^ whereas our cohort had a mean age of 66 (SD:6) years old. The relatively low mean age in our sample may have contributed to a relatively low prevalence of LATE (8.2% for all LATE groups combined), and previous studies have reported higher percentages in older clinico-pathological cohorts (estimated to be 25%–40% in ≥85 years of age).^10^ Of interest, however, we observed that individuals identified as Probable LATE in our memory clinic sample were not older than those in the AD group and were younger than individuals with Possible LATE and LATE-AD (eTable 5). This hints toward a more nuanced association between age and LATE, which should be examined in cohorts encompassing a sufficiently broad age range to allow direct assessment of age effects on the prevalence of clinically defined LATE. In line with a previous investigation,^48^ we observed that, at baseline, amygdalar atrophy in Probable LATE was more pronounced compared to AD, and the ITH ratio indicated pronounced limbic-predominant atrophy. Given that visual reads of medial temporal atrophy were used as a classification criterion in our operationalization of LATE, there is some degree of circularity in these results. However, it does highlight that these specific MRI measures may be of utility for classifying individuals with Probable LATE.

In contrast to the Probable LATE group, the Possible LATE group showed similar clinical trajectories and mortality rates compared to AD. As also alluded to in the term Possible LATE, these results suggest that this group is not only neurobiologically (i.e., partly comprised A+ individuals) but also clinically less distinct from “pure” AD. Our baseline atrophy measures did reveal that ITH ratios were different between individuals with Possible LATE and AD. Together with previous findings on the same effect,^33^ this indicates that the ITH ratio metric could be a sensitive marker of LATE-NC, sensitive enough to distinguish Possible LATE from AD. Of note, the Possible LATE group comprised both individuals with Aβ-positive but tau-negative biomarkers (A+T−; possibly indicating incipient AD) and individuals with missing Aβ biomarkers (A_missing_; possibly indicating undetected AD). We show that the A+T− and A_missing_ subgroups show different clinical profiles and trajectories. This highlights the importance of incorporating biomarkers into criteria for LATE, suggesting that individuals with missing biomarkers may not fit well into the same category as individuals with a known biomarker profile, which might be considered in future iterations of clinical criteria for LATE. A similar refinement of biomarker categories might also be pertinent to the Probable LATE group, where biomarkers were implemented to rule out AD pathology. In the current clinical criteria, this was based only on Aβ-biomarkers, whereas an A−T+ profile was eligible for classification into the Probable LATE group.^10^ We showed that the A−T+ subgroup showed differential clinical trajectories compared to the rest of the Probable LATE group, suggesting that a further refinement of the clinical criteria might also be needed here.

Aside from the classification of Probable LATE and Possible LATE, the current clinical criteria also allow for a group for whom the clinical symptoms are likely related to co-occurring LATE-NC and AD pathology.^1^ In the present study, this LATE-AD group had faster cognitive decline and higher mortality rates compared to “pure” AD, which is in line with previous reports.^1,8,9,11,13,14^ This accelerated clinical decline is likely driven by the additive burden of both LATE-NC and AD pathology.^1,2,9,12,49,50^ Aside from the clinical effects of LATE-AD, we also observed that this group shows greater atrophy within an AD-signature cortical thickness mask. Although we did not detect accelerated rates of atrophy after our screening visit, this pronounced cortical atrophy in individuals with LATE-AD suggests synergistic pathologic effects of LATE-NC and AD pathology on downstream neurodegeneration. Identifying LATE-NC in those who also have AD pathology is currently a major challenge because of the lack of validated molecularly specific biomarkers for TDP-43. What's more, by allowing the co-occurrence of LATE-NC and AD pathology, one can not rely on excluding AD biomarkers in this context. Instead, one must rely solely on clinical and radiologic features, which is further complicated because these overlap between LATE and AD. The designation of Possible LATE-AD, which was not used in this text to avoid confusion with the Possible LATE group, but is the correct classification, therefore, remains prudent.

A key strength of this study is that we apply the newly developed clinical criteria for LATE in a large memory clinic cohort, closely resembling the intended real-world clinical use of these criteria. This intended use also guided our decision to classify LATE based on MRI criteria using visual read measures, which are commonly performed in clinical practice and do not require additional MRI processing. Our study also has several limitations. First, due to the structure of the data source, the ADC, which prioritizes comprehensive initial screening over extensive follow-up assessments, we had to operationalize the Wolk et al.^10^ criteria in a way that did not account for the hypothesized indolent course of LATE. However, we were able to demonstrate this progression pattern after classification and show that adapting the criteria to accommodate cross-sectional data results in valid derivative criteria that can be used in memory clinic settings. Second, and similarly, our mean age of 66 (SD 6) years old did not allow us to apply the criteria of “Generally >75 years old,” which was listed as not required in the consensus criteria.^10^ Instead, we opted to assess age differences between the groups and found that, in our sample, older age was not related to a higher chance of LATE. The observed differences in clinical trajectories between the groups, without applying an age criterion, highlight that our operationalization of the criteria produces a clinically meaningful classification. Third, our study lacked sufficient tau-PET or fluorodeoxyglucose (FDG)-PET data to incorporate these biomarkers into our operationalization of 2 key supportive features for diagnosing LATE. Fourth, we did not detect any differences in longitudinal atrophy between our diagnostic groups. This is likely due to our limited sample (N = 250 across all groups) of individuals with longitudinal MRI, highlighting the need to assess these effects in future large-scale investigations. Last, we operationalized and tested clinical criteria for LATE that aim to predict the chance that LATE-NC is the underlying cause for clinical symptoms.^10^ However, these criteria have not been thoroughly neuropathologically validated, and this needs to be considered when drawing conclusions regarding clinical vs neuropathology associations.

LATE presents a significant challenge in neurodegenerative disease research, due to the lack of molecularly specific biomarkers for TDP-43 pathology and clinical and radiologic similarities to AD. As developments in disease-modifying treatments against AD advance, the need to differentiate individuals with LATE and AD pathology and assess treatment efficacy in individuals who might have (comorbid) LATE-NC becomes imperative. Clinical criteria, such as discussed here, provide an essential framework for identifying potential LATE cases in vivo, yet further advancements in biomarker development are necessary to refine diagnosis and enhance our understanding of the clinical and radiologic manifestations of LATE-NC. There have been exciting advances in biofluid biomarker research, particularly the detection of TDP-43 in plasma extracellular vesicles,^51^ but valid and reliable biomarkers for detecting LATE-NC during life are still not available. LATE criteria will, therefore, continue to rely on clinical and radiologic features to obtain a probabilistic estimate of the likelihood of LATE-NC. This study highlights the clinical applicability of these types of criteria, supporting their use as a new diagnostic classification in memory clinics. We hope that further research will continue to advance the field, ultimately leading to accurate and early diagnosis of LATE.

## Glossary

Aβ: β-amyloid
AD: Alzheimer disease
ADC: Amsterdam Dementia Cohort
GCA: global cortical atrophy
HR: hazard ratio
ITH ratio: inferior temporal thickness/hippocampus volume
LATE: limbic-predominant age-related TDP-43 encephalopathy
LATE-NC: LATE neuropathologic change
LMM: linear mixed model
MCI: mild cognitive impairment
MMSE: mini-mental state examination
MTA: medial temporal atrophy
PA: posterior atrophy
ROI: region-of-interest
TDP-43: TAR DNA-binding protein 43
